# Utilization of youth friendly services and associated factors among youth in Harar town, east Ethiopia: a mixed method study

**DOI:** 10.1186/s12913-016-1513-4

**Published:** 2016-07-17

**Authors:** Aboma Motuma, Thomas Syre, Gudina Egata, Abera Kenay

**Affiliations:** School of Nursing and Midwifery, College of Health and Medical Sciences, Haramaya University, P.O. Box. 235, Harar, Ethiopia; Department of Public Health, College of Health and Medical Sciences, Haramaya University, P.O. Box. 235, Harar, Ethiopia

**Keywords:** Ethiopia, Reproductive health, Utilization, Youth friendly services

## Abstract

**Background:**

Youth friendly services are designed to make health services accommodate the unique needs of youth. Nevertheless, in developing countries like Ethiopia, the level of knowledge about the use of these services is limited. The main aim of this study was to assess the extent of youth friendly service utilization and the associated factors among the youth.

**Methods:**

A community based- cross sectional quantitative study design supplemented with qualitative inquiry was used from January to February 2011. Data were collected from a random sample of 845 youth using a pretested structured questionnaire. Qualitative data were collected through interview guides. Odds ratios, along with 95 % confidence level, were estimated to measure the strength of association between the study variables using multivariable logistic regression. Level of statistical significance was declared at *p*-value less than 0.05. Thematic analysis was used to analyze the qualitative data.

**Results:**

Nearly 64 % of the youth had already utilized youth friendly services at least once at the time of the survey. In multivariable logistic regression analysis, using friends [AOR = 3.65, 95 % CI (1.81,7.32)], health care providers [AOR = 3.27, 95 % CI (1.18,9.00)], and schools [AOR = 1.79, 95 % CI (1.00,3.19) as source of information, and having knowledge about the youth friendly services [AOR = 2.77,95 % CI (1.93,3.96)] were significantly associated with the utilization of youth friendly services. In contrast, being daily laborer and private worker by occupation [AOR = 0.12, 95 % CI (0.05, 0.92)], having negative perception about counseling [AOR = 0.50, 95 % CI (0.31–0.80)], about reproductive health services [AOR = 0 .13, 95 % CI (0.04–0.46)], and about youth friendly service providers [AOR–0.02, 95 % CI (0.08–0.50)] negatively influenced the outcome variable.

**Conclusions:**

The utilization of youth friendly services is moderate in this study. Getting youth related services information from different sources and being knowledgeable about the services have increased the utilization of the services. Efforts should be made by all relevant stakes to create conducive environment for the youth through training of the youth service providers, particularly for those who work in the government institutions, and strengthening of the awareness creation strategies among the youth to increase the utilization of the services.

**Electronic supplementary material:**

The online version of this article (doi:10.1186/s12913-016-1513-4) contains supplementary material, which is available to authorized users.

## Background

According to the World Health Organization (WHO), youth refer to people aged between 15 and 24 years and are characterized by unique physical, psychological, social, and emotional changes that put their life at high risk. They account for 22 % of the total population of Ethiopia [1, 2].

Since the 1994 International Conference on Population Development (ICPD), countries have been encouraged to adopt programs that safeguard the youth’s privacy, confidentiality, respect and informed consent [3]. Such services that are able to meet such expectations are known as youth friendly services (YFS) and are designed to improve care for the youth [4]. The services include counseling, family planning, Voluntary Counseling and Testing (VCT) and treatment of Sexually Transmitted Infections (STIs) [5]. These services should be accessible, acceptable and appropriate for the youth. They should be rendered in the right place with reasonable price, sometimes for free when necessary, and delivered in the right style to be acceptable to the young people and are effective, safe and affordable.

In pursuing the Reproductive Health (RH) agenda and to meet its youth’s needs by 2015, the government of Ethiopia has developed a 10 year National Adolescent and Youth Reproductive Health Strategy (NAYRHS). Despite these initiatives, the YFS utilization among the youth is still low and faces a lot of challenges related to the sensitive nature of adolescents’ sex and sexuality and their perception of model health care delivery. Thus, access to and utilization of YFS has become a primary concern of Sexual and Reproductive Health (SRH) rights in the country [6, 7].

Many youth are less informed, less experienced, and less comfortable in accessing health services for RH than are adults. The youth often lack basic RH information, knowledge, and access to affordable and confidential health services for RH. They do not feel comfortable in discussing RH matters with their parents [8, 9]. The main barriers relating to young people’s lack of access to health services included cost, inconvenient time or location of youth related services [10], lack of knowledge about the available services [11], confidentiality [12] and perceived or real fear about reactions of health care providers [4, 13].

The WHO has estimated that 70 % of the premature deaths among adults are largely due to behaviors initiated during adolescence [14]. In addition to this, many scholars have indicated that 17 % of young women and 14 % of young men aged between 20 and 24 years were sexually active by the age of 15 years [4]. However, in Ethiopia, utilization of family planning services in the existing health care delivery system by young people was very low and a high rate of unintended pregnancy and abortion complications occur. For instance, 67.2 % of those seeking treatment for incomplete abortion were aged under 24 years [5]. Another major health threat affecting the young people was Reproductive Tract Infections (RTIs) including HIV/AIDS. It is estimated that over a quarter (26 %) of the HIV positive population in Ethiopia are aged between 10 and 24 years [15].

In 2004, the ministry of youth, sports and culture of Ethiopia developed a national youth policy to address the multi-lateral youth problems and to coordinate efforts of different stakeholders. Of the ten issues the policy tries to incorporate are youth and health, and youth and HIV/AIDS [15].

Despite the fact that youth are suffering from substantial negative youth related health consequences, studies on the level of YFS utilization and the associated factors are very limited in Ethiopia. Thus, this study aimed to assessing the level of utilization of YFS and the associated factors among the youth in Harar town, east Ethiopia. The results of this study will be used by the policy makers and the youth health planners to design appropriate youth targeted interventions in the country.

## Methods

### Study setting and design

A community based cross-sectional quantitative study design supplemented with qualitative inquiry was used to assess the extent of youth friendly service utilization and the associated factors among the youth from January to February 2011 in Harar town, east Ethiopia. Harar is located at a distance of 525 Km away from Addis Ababa, the capital city of Ethiopia. The population of the town was estimated to be 107,531 with an estimated youth population of 23, 657 in 2010 prior to the survey [2]. The town is divided into six districts and 19 kebeles (the smallest administrative unit in Ethiopia). In the town, there are three government hospitals, one university teaching hospital, two private hospitals, eight health centers, and a branch of Family Guidance Association of Ethiopia (FGAE) youth center and model clinic.

The study was supplemented by qualitative approach to explore in more detail the underlying behaviors, attitudes, perceptions, and culture [16] that might impact YFS and due to the sensitivity of the subject matter and complexity of genuinely reporting youth related SRH issues [17] from youth and health care providers perspectives [18].

### Sample size determination and sampling procedure

The sample size for the quantitative study was calculated using a single population proportion formula with the following assumptions: utilization of YFS by youth to be 50 %, a 5 % margin of error, 95 % confidence level, and a design effect of 2, making the possible maximum sample size of 845 youth for the study..

For the qualitative inquiry, six in - depth interviews (IDIs) were conducted, 3 with YFS providers working at government institutions and 3 with service providers in the Harar branch FGAE. Additionally, four Focus Group Discussions (FGDs), two with female youth and two with male youth, were conducted.

A multistage sampling procedure was used to select the study participants from all youth aged 15–24 years and living in the town. The town was stratified into six strata, using the existing division of the district. From each district, one kebele was selected by a lottery method. The study samples were proportionally allocated to the youth population size of each kebele. In order to develop a sampling frame, a baseline enumeration of the total households in each kebele was conducted. Then, a simple random sampling technique was used to select the eligible households from the sampling frame. The youth who were residents of the selected kebeles for at least six months were included in the study. The participants for IDIs and FGDs were selected purposively from the study area.

### Measurements

The quantitative data were collected using an interviewer administered pretested structured questionnaire which was adopted from different literatures. The questionnaire contained the youth’s socio-demographic characteristics, knowledge about and attitude towards YFS, and level of utilization and the factors associated with YFS. It was initially prepared in English and then translated into “Amharic”, the local language, and finally retranslated back into English by fluent speakers of both languages.

The qualitative data were collected using semi-structured interview guides designed for YFS providers and the youth themselves (Additional file 1: Table S1 and Table S2). Discussions were held with FGDs and IDIs discussants in Amharic until the point of saturation was reached. The discussions were tape recorded with permission from the participants. The transcribed data were compared with the notes taken during IDIs and FGDs.

Utilization of YFS was the outcome variable in this study. It was defined as using any of the basic RH services such as counseling services, HIV testing, contraceptive use, STIs diagnosis and management, pregnancy testing, post-natal care and abortion care services and other youth targeted non health related services such as library and internet services in the last twelve months before the survey. Utilization of YFS was coded as “1” and “0” otherwise for further analysis.

The explanatory variables included socio-demographic characteristics such as age, religion, education, occupation, income, source of information for YFS, knowledge about YFS, importance of RH for youth, importance of counselling for youth, welcoming behavior of service providers, importance of contraceptives, and welcoming environment of service institution. Knowledge about YFS was calculated on the basis of the scale from 0 to 5 for five knowledge questions. The Mean knowledge was calculated and the cut-off point for determining knowledge was developed based on literature. Values below the mean were coded as “1” while values above the mean were coded as “0” for further analysis. In this study, far distance was understood as travelling greater than an hour to access the YFS while inconvenient location was understood as location of YFS center which can easily expose the youth to adult population in the area, although the service is accessible, that forces them not use the service.

In order to maintain the quality of the data, a properly designed data collection instrument was used. An intensive training was given for both the data collectors and the supervisors for 3 consecutive days. The questionnaire was pretested on 5 % of the study population, outside the study area, who had similar characteristics with participants of this study, and the necessary modifications were made while considering its content validity before the actual data collection. Six well trained and experienced nurses who had had experience on research data collection gathered the quantitative data. Four supervisors (two males and two females) who were holders of Masters in Public Health (MPH) supervised the process of the quantitative data collection. Additionally, they were involved in the qualitative data collection under the close supervision of the investigators. The FGDs and IDIs discussion with female youth and YFS providers was moderated by female supervisors, whereas male supervisors did with the men to facilitate easy and free discussion.

### Data processing and analysis

The data were entered onto Epi Data Version 3.1 computer software, after checking for completeness. They were then exported to statistical software package for social sciences (SPSS) Version 16 computer software for further analysis. Descriptive statistics such as frequencies, proportions, and numerical summary measures were used to describe the data. Multicollinearity test was carried out to see the correlation between each explanatory variable, using Variance Inflation Factor (VIF) and tolerance test. The values for both tests were within the normal cut-off points. Bivariate analyses were used to assess the association between the outcome variable and each independent variable. All variables that showed significant association during the bivariate analyses were entered to multivariable logistic regression to control for all possible confounders, using enter method. The Odds ratio, along with 95 % confidence level, was estimated to identify the factors associated with YFS utilization and the level of statistical significance was declared at *p*- value less than 0.05.

The qualitative data were transcribed verbatim, typed in word, and translated back into English by the investigators and analyzed using thematic analysis. The main thematic areas from the qualitative data were identified, coded, and significant quotes were also noted in the text.

## Results

### Socio-demographic characteristics

All the randomly selected participants (*n* = 845) have responded to the questionnaire making a response rate of 100 %. Nearly half of the respondents (51.4 %) were males. The mean (±SD) age of the respondents was 18.7 (±2.8) years, with 62.1 % of them in the age range of 15–19 years. Most of them were living with their parents (71.5 %) and single (81.6 %), and 66.4 % were in-school youth, of whom 42.3 % were grade 9 and 10 students (Table 1).

**Table 1:** Socio-demographic characteristics of youth in Harar town, east Ethiopia, 2011(*n* = 845)

Characteristics		Frequency	Percent
Age	15–19	525	62.1
	20–24	320	37.9
Sex	Male	434	51.4
	Female	411	48.6
Religion	Orthodox	425	50.3
	Muslim	331	39.2
	Protestant	78	9.2
	Catholic	8	0.9
	Others	3	0.4
Educational status	Illiterate	31	3.7
	Primary	284	33.6
	Secondary	358	42.3
	12 and above	172	20.4
Marital status	Single	689	81.6
	Married	138	16.3
	Divorced	6	0.7
	Widowed	12	1.4
Ethnic group	Oromo	340	40.2
	Amhara	328	38.8
	Harari	47	5.6
	Guraghe	66	7.8
	Tigre	46	5.5
	Others	18	2.1
Occupation	Student	560	66.3
	Employed	58	6.9
	Merchant	31	3.7
	Housewife	81	9.5
	Unemployed	55	6.5
	Others	60	7.1
Monthly income	No income	627	74.2
	Below 300 birr	34	4.1
	300–900 birr	101	11.9
	900 birr and above	54	6.4
	Don’t know	29	3.4
School status	In school	561	66.4
	Out school	284	33.6
Living arrangement	Married	132	15.6
	Live with boy/girl friend	109	12.9
	With parent/s	604	71.5
Have child	Yes	112	13.3
	No	733	86.7
Number of child(*n* = 112)	1–2 children	75	67
	3–4 children	24	21.4
	4 and above children	13	11.6

### Knowledge and attitude towards youth friendly services

**Fig. 1:**
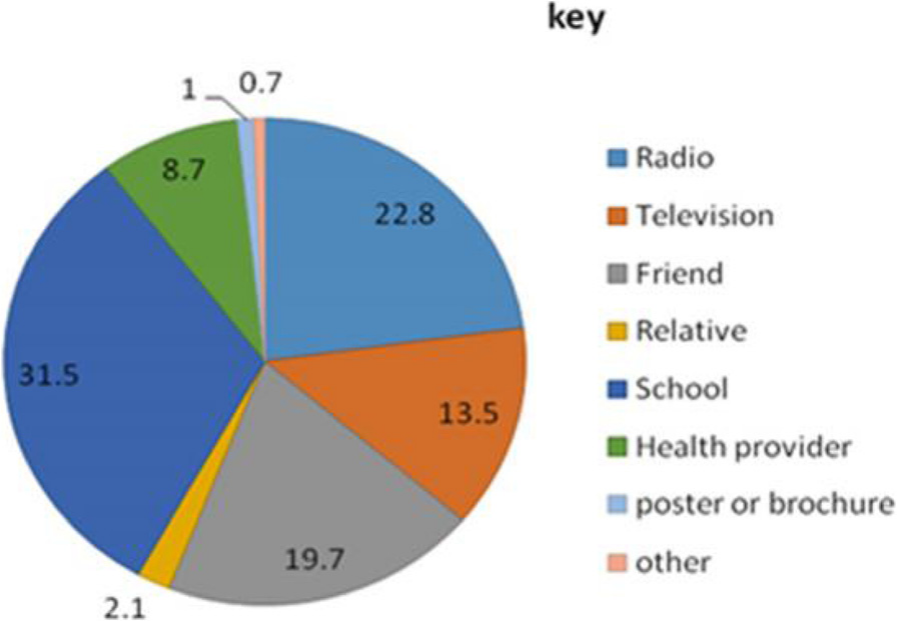
Source of YFS information among youth in Harar town, Ethiopia, 2011

Most of the respondents, 612(72.4 %), had information about YFS mainly from school teachers (31.5 %) and radio (22.8 %) (Fig. 1). Besides, 749(88.6 %) of them believed that youth friendly services are necessary for the youth.

The most important component of youth friendly services reported by the youth were STIs including HIV/AIDS (57.4 %) and counseling services (44.7 %). Similarly, they reported STIs (54.9 %) and counseling (37.2 %) as the actual YFS given at the health institutions. Nearly 70 % of the participants reported that the youth should get important information education and communication (IEC) on RH at the age of 15 years or older. The major RH problems reported by the youth were unintended pregnancy (72.4 %), STIs/HIV/AIDS (49.8 %), unplanned sexual practice (43.5 %), and abortion (34.5 %). Three in four (74 %) of the youth indicated that the youth should involve in addressing their own RH problems.

The mean score knowledge of YFS on five points scales was 0.316 (± SD = 0.465). From all the respondents, only 267(31.6 %) were knowledgeable about YFS given at the health institutions. About one in five (18.1 %) of the youth never mentioned any YFS whereas half (50.3 %) of them reported only one component. Responses to the commonest diseases acquired through sexual intercourse included HIV/AIDS (91.1 %), gonorrhea(50.5 %),syphilis(21.9 %),and chancroid (15.7 %).

Abstaining from sex (55.9 %) followed by using condom (53.1 %), faithfulness to partner (37.5 %) and avoiding sex with commercial sex workers (6.2 %) were the major modes of preventing STIs reported by the youth. More than one in three respondents (69.2 %) had positive attitude towards YFS.

### Utilization of youth friendly services

Though 82.2 % of the youth reported that they know where YFS are delivered, only 63.8 % of them reported that they used the services at least once in the last twelve months preceding the survey. Many of the FGD discussants indicated that the youth visit the YFS centers to get counseling services, education and information on RH services and library services. Almost all the participants strongly believed that the existence of youth centers with some recreational activities such as internet services, sport activity and information centers (library services) made youth feel good about seeking youth friendly services.

The FGAE model clinic was the more visited institution (64.5 %) compared with the public institutions. About 65.8 % of the visits were for the first time. Most of the visits were for RH services (96.1 %), whereas very few were for library and recreational services (3.9 %), and most of the youth visited the YFS providing facilities to get counseling (59.7 %), services on STIs (31 %), for contraceptive services (14.3 %), IEC on RH services (12.4 %), pregnancy related services(6.3 %),and post abortion care(1.3 %.). In this study, 36.2 % of the youth did not use any YFS mainly because they did not know the place (42.8 %), were living far from the facility (18.7 %), were healthy (15.1 %), felt the location was inconvenient (11.8 %), thought the services were of poor quality (11.8 %), and found the opening time inconvenient (3.3 %).

### Factors associated with utilization of youth friendly services

In the bivariate logistic regression analyses, being in the age group of 15–19 years [COR = 0.68, 95 % CI (0.51,0.92)], being a muslim [COR = 0.71, 95 % CI (0.53–0.96)], being illiterate [COR = 0.28, 95 % CI (0.12,0.61)] and being 1- 8^th^ grade [COR = 0.50, 95 % CI (0.33,0.76)], having negative perception about the importance of RH for youth [COR = 0.34, 95 % CI (0.17–0.68)] and not knowing RH itself [COR = 0.08, 95 % CI (0.04–0.16)], having negative perception about the importance of contraceptives [COR = 0.64, 95 % CI (0.43–0.94)] and counseling for youth [COR = 0.67, 95 % CI (0.49–0.91)], unfavourable attitudes of youth towards the behavior of YFS providers [COR = 0.50, 95 % CI (0.31–0.80)] and not knowing about their behavoiur itself [COR = 0.16, 95 % CI (0.11–0.22)] and unfavourable attitudes of youth towards the conduciveness of health service institutions [COR = 0.45,95 % CI (0.28–0.72)] and not knowing about them [COR = 0.16, 95 % CI (0.11–0.24)] were negatively associated with utilization of youth friendly services. However, being employed [COR = 2.72, 95 % CI (1.35,5.51)], having income level of 300–900 ETB [COR = 2.56,95 % CI (1.53,4.29)], using health care provider as source of information for YFS [COR = 2.62, 95 % CI (1.09,6.31)], and having knowledge about YFS [COR = 2.78, 95 % CI (1.99,3.90)] were positively associated with the outcome variable (Table 2).

**Table 2:** Factors associated with utilization of youth friendly services among youth in Harar town, 2011

Characteristics	Utilized YFS		COR(95 % CI)	AOR (95 % CI)
	Yes	No		
Age				
15–19 20–24	310(59) 221(69.1)	215(41) 99(30.9)	0.68(0.51,0.92)* 1.0	0.65(0.36,1.18) 1.0
Religion				
Orthodox	306(67.3)	119(32.7)	1.0	1.0
Muslim	197(59.3)	134(40.5)	0.71(0.53–0.96)*	1.00(0.62,1.63)
Protestants	51(65.4)	27(34.6)	0.91(0.55–1.52)	0.82(0.40,1.69)
Catholic	4(50)	4(50)	0.48(0.12–1.97)	0.57(0.05,5.78)
Other	1(33.3)	2(67.7)	0.24(0.02–2.70)	0.55(0.00,34.76)
Educational Status of youth				
Illiterate	13(41.9)	18(58.1)	0.28(0.12,0.61)*	1.57(0.22,11.01)
1–8 grade	161(56.7)	123(43.3)	0.50(0.33,0.76)*	1.11(0.55,2.22)
9–12 grade	241(67.3)	117(32.7)	0.79(0.53,1.18)	1.46(0.79,2.72)
Above 12	124(72)	48(28)	1.0	1.0
Occupational status of youth				
Students	357(63.8)	203(36.2)	1.0	1.0
Employed	48(82.8)	7(17.2)	2.72(1.35,5.51)*	0.66(0.14,3.02)
Merchant	24(77.8)	7(22.6)	1.95(0.82,4.60)	0.39(0.07,1.92)
Housewife	47(58)	34(42)	0.78(0.48,1.26)	1.50(0.52,4.29)
Unemployed	28(51)	27(49)	0.59(0.33,1.02)	1.06(0.35,3.19)
Other^a^	33(58.3)	25(41.7)	0.79(0.46,1.36)	0.12(0.05,0.92)*
Income (ETB) of youth				
No income	384(61.2)	243(38.8)	1.0	1.0
Below 300 ETB	20(58.9)	14(44.1)	0.80(0.40,1.60)	3.69(0.82,16.5)
300–900 ETB	81(80.2)	20(19.8)	2.56(1.53,4.29)*	7.42(1.99,27.68)
Above 900 ETB	37(68.5)	17(31.5)	1.37(0.75,2.50)	3.19(0.73,13.96)
Do not know	18(62.1)	11(37.9)	1.03(0.48,2.13)	0.98(0.31,3.01)
Source of information for YFS				
Radio	100(71.4)	40(28.6)	1.0	1.0
Television	66(79.5)	17(20.5)	1.55(0.81,2.96)	1.91(0.90,4.03)
Friends	99(81.8)	22(18.2)	1.80(0.99,3.24)	3.65(1.81,7.32)*
School	137(71)	56(29)	0.97(0.60,1.58)	1.79(1.00,3.19)*
Health care provider	46(86.8)	7(13)	2.62(1.09,6.31)*	3.27(1.18,9.00)*
Poster/brochure	5(83.3)	1(16)	2.00(0.22, 17.65)	3.16(0.32,31.08)
Other	3(75)	1(25)	1.20(0.12,11.88)	1.17(0.10,13.57)
Knowledgeable about YFS				
Knowledgeable	210(78.7)	57(21.3)	2.78(1.99,3.90)*	2.77(1.93,3.96)*
Not knowledgeable	329(56.9)	249(43.1)	1.0	1.0
RH is important for youth				
Yes	516(68.8 %)	235(31.2 %)	1.0	1.0
No	14(21.9 %)	50(78.1 %)	0.34(0.17–0.68)*	1.74(0.20–15.13)
Do not know	9(12.5 %)	63(87.5 %)	0.08(0.04–0.16)*	0.13(0.04–0.46)*
Counseling is important for youth				
Yes	240(72.7 %)	90(27.3 %)	1.0	1.0
No	268(64.0 %)	151(36.0 %)	0.67(0.49–0.91)*	0.50(0.31–0.80)*
YFS providers are welcoming				
Yes			1.0	1.0
No			0.50(0.31–0.80)*	2.07(0.78–5.48)
Don’t know			0.16(0.11–0.22)	0.02(0.08–0.50)*
Contraceptive is important for youth				
Yes	124(75.2 %)	41(24.8 %)	1.0	1.0
No	384(65.8 %)	200(34.2 %)	0.64(0.43–0.94)*	0.82(0.46–1.48)
Health service institutions are conducive				
Yes	435(74.4 %)	150(25.6 %)	1.0	1.0
No	47(56.6 %)	36(43.4 %)	0.45(0.28–0.72)*	0.68(0.28–1.61)
Don’t know	57(32.2 %)	120(67.8 %)	0.16(0.11–0.24)*	0.82(0.29–2.32)

*ETB* Ethiopian birr, *COR* crude odds ratio, *AOR* adjusted odds ratio

*p- value ≤ 0.05, ^a^ private worker and daily laborer

In multivariable logistic regression analysis, using friends [AOR = 3.65, 95 % CI (1.81,7.32)], health care providers [AOR = 3.27, 95 % CI (1.18,9.00)] and schools [AOR = 1.79, 95 % CI (1.00,3.19) as source of information for YFS and having knowledge about YFS [AOR = 2.77, 95 % CI(1.93,3.96)] were significantly associated with utilization of youth friendly services. In contrast, belonging to other categories by occupation (private worker and daily laborer) [AOR = 0.12, 95 % CI (0.05, 0.92), not knowing the importance of RH for youth [AOR = 0.13, 95 % CI (0.04–0.46)], and not knowing whether YFS providers are conducive or not to youth [AOR = 0.02, 95 % CI (0.08–0.50)] have negatively affected the YFS utilization (Table 2).

The youth who were knowledgeable about YFS were nearly three times more likely to utilize the service compared with their counterparts. The respondents who heard about YFS information from their friends, health care providers, and schools were nearly 4 times, 3.3 times and 2 times more likely to utilize YFS, respectively compared with their counterparts.

Supporting these findings, most of the discussants indicated that the adolescents are reluctant and uncomfortable to discuss RH issues. In most cultures, open discussion of RH issues with parents and significant others is minimal due to the conservative cultural and religious practices. Because of this, the youth do not have adequate information about their RH needs and problems. Most of the discussions between family and the adolescents occur only after certain RH problem has occurred. Most parents are ill-prepared, uncomfortable or awkward in discussing RH issues with their children. This will make adolescents lack knowledge and skills to make rational decision and seek contraceptive or other RH services. Throughout the discussion, culture was repeatedly raised as a factor that prevented the youth from acquiring essential youth SRH services. For example, a 17- year female discussant said “*In our culture if you ask about sex related information, others will perceive as if you were already in the process”.*

In this study, some of the factors that negatively influenced the utilization of youth friendly reproductive health services were being daily laborer and private worker [AOR = 0.12, 95 % CI (0.05, 0.92)], having negative perception about counseling [AOR = 0.50, 95 % CI (0.31–0.80)], about reproductive health services for the youth [AOR = 0 .13, 95 % CI (0.04–0.46)],and about YFS service providers [AOR = 0.02, 95 % CI (0.08–0.50)]. One of the major reasons stated in the discussion by the youth for not using the services at the health institutions was feeling discomfort by the conditions of the centers or the attitude of the service providers. Many of these perceptions were resulted from second hand information or general public attitude. For instance, a 21 year- old male discussant who visited the facility for contraceptive described “*You know, if you go for family planning, they will ask you about your marital status”. A* 22 year- old female discussant also described the experience of her friend’s discomfort about working time as follow: *“She went to the youth center to get emergency contraceptive. But the center was closed. Had she been successful, she will be in university today since her pregnancy could have been avoided”.* Moreover, a 20 years old male discussant indicated that most of government centers lack separate youth clinics saying *“When you go to hospitals for services, you may meet your parents there. I remember my friend who met her mother in a clinic”.*

Other youth indicated the importance of establishing recreational/sport facilities and libraries/internet within the youth center. They also indicated that the youth model clinic run by the FGAE has such facility, unlike other government centers. The presence of such facilities will decrease fear of public attitude, beyond being source of information. A 22 year- old female discussant said, “*I can go to the center taking my note books with me. There I can get the health service I need. But the public perceive as I went for library. This is impossible in public hospitals “. It was* also indicated that such facilities will improve the understanding of the youth about the common RH problems, aware of communication skills, and learn the experience of other youth.

On the other hand, the YFS providers explained that although youth’s RH needs are immense, there are obstacles to access health services. The service providers reported that when the youth reached the centers, passing all obstacles, good decision should be made in support of them. The knowledge and belief of providers play a large role in the kind of information the youth will obtain or the service they will utilize. The sex of the service providers, receiving training or not, judgmental attitude toward adolescent sexual activity and up to date knowledge on such issues were raised as affecting the utilization of the YFS.

Similarly, other respondents of the in-depth interview from public institutions explained that there were no specifically designed SRH services for the youth in their institution. They explained that SRH services are offered for youth without giving them special attention, treating them as adult clients.

Similar to the finding from FGDS with the youth, there are perceptions that giving RH service for adolescents is difficult. For example, according to a male respondent from government hospital, “*Family planning should not be given to adolescents; they should be educated only because family planning is good only for married couples. Instead, he suggested discussion about abstinence until marriage*”. Moreover, according to a female respondent from another government hospital, “making decision is sometimes difficult, *imagine what you would do when you are asked for family planning by a 13 years old female. How I can give this girl a contraceptive*?”.

Additionally, the respondents of the in-depth interview were asked whether they have been given training on YFS and approaching the youth politely. The respondents from the family guidance association reported that they have taken such trainings and are confident that they could respond to the needs of the youth. However, the respondents from the government hospitals indicated that they treat the youth like any adult clients. They also reported that they are using their experience to provide YFS but feel not comfortable. One respondent from the government institution said, “…. *I have not been offered on the job training on how to provide YFS. Just I am doing what I feel correct from my experience, though I don’t feel comfortable. But I am reading and trying to be a good provider as much as I can….”*. Other providers also reported lack of confidence about responding to the needs of youth. A nurse from hospital claimed “…*I feel incompetent because sometimes youth ask me so many questions which I can’t fully answer. So I feel even defeated…”*

Respondents from both government and non-governmental centers indicated the presence of national guidelines to work on the youth but only those from the non-government centers reported that they are using it.

## Discussion

In this study, nearly 64 % of the youth utilized YFS at least once at the time of the survey. In multivariable analysis, using friends, health care providers, and schools as source of information for YFS and having knowledge about YFS were positively associated with the utilization of YFS. In triangulation most of the qualitative findings are in support of the quantitative findings. These key findings of the study are worthwhile and could enable policy makers and health planners to improve youth targeted interventions in the study setting in particular and in the country at large.

The level of YFS utilization in this study was 63.8 %. This finding is higher than the ones reported from similar studies conducted in other parts of Ethiopia [8, 9, 19–23]. This variation could be attributed to difference in size and settings of the study. The extent of using YFS observed in this study is said to be unsatisfactory. Supporting this unsatisfactory coverage, less than half of the youth mentioned lack of awareness about the location of YFS facilities as a major reason for not utilizing the services. Therefore, more attention is needed from all relevant stakeholders to improve the utilization of YFS by curbing the reasons.

There are differences between and within settings concerning the level and type of YFS utilization. In this study, nearly 60 % of the study participants received reproductive counseling, 31 % got services on STIs, and about 14.3 % of them visited the center for contraceptive services. The findings on counseling and STIs services are greater than the result of previous similar study conducted in the country while the percentage observed for contraceptive services is similar with the same study [8].

The respondents reported that they visited FGAE model clinic and youth centers more than they did public institutions. This finding is different from the finding in Addis Ababa, where many of the youth visited public institutions, but similar to the finding from Jimma town. As it has been noted in the interview, this could be related to having trained providers and library/internet services or other recreational centers in the non-governmental organizations (NGOs) youth centers. Moreover, as it was indicated by the FGD discussants, most of the government centers lack separate youth clinics, which could have resulted in fear of seeking the services. This finding is comparable with other findings whereby being embarrassed or fear not to be seen by parents or someone else who knows the youth was reported for not using YFS [8, 9, 24]. However, exceptionally there are specifically designed youth centers run by the government in Addis Ababa, which might have similar or better facilities compared with the FGAE’s youth centers.

One of the major reasons for not using YFS among the youth was lack of awareness about the location of YFS facilities, which accounts nearly for 43 %. The proportion is higher than the findings from other previous studies [8, 9]. This could be attributed to the extent of the promotion of the centers and the services in the study areas. Another possible explanation could be the difference in the age profile of the respondents included in this study and previous studies. This could as well affect sexual status, educational status or other socio- demographic characteristics of the respondents, which might in turn affect the entire utilization of RH services.

Different sources of information for YFS influenced its utilization among the youth. The utilization of YFS was higher among the youth who received information from health care providers, friends and schools, and who had knowledge of YFS in our study. This finding is comparable with the finding from elsewhere in the country [8] and might strengthen the general expectation that knowledge is of paramount importance before practice.

In this study, like in other similar studies [8, 9, 14],.the chance of using the services was estimated to be less among the respondents who perceived that reproductive health services and counseling are not important for the youth, who have engaged in sub-standard occupation, and who lack awareness on the welcoming nature of the service providers. This implies that a lot has to be done on awareness creation about the nature of YFS and support the youth so that they could pay due attention to a pool of YFS services and make use of the available services.

This study could have the following limitations: Firstly, the study related to reproductive issues is much sensitive and might result in social desirability bias, which could underestimate or overestimate an outcome of interest. Secondly, some reproductive related questions were asked to track past experiences of the youth, which might be subject to recall bias.and thus could compromise the findings of the study. However, careful attention was given to the study in advance by the researchers to maintain the quality of the data.

## Conclusions

The utilization of youth friendly services is moderate in this study. Getting youth related services information from different sources and being knowledgeable about the services have increased the utilization of the services. Efforts should be made by all relevant stakes to create conducive environment for the youth through training of the youth service providers, particularly for those who work in the government institutions, and strengthening of the awareness creation strategies among the youth to increase the utilization of the services.

## Abbreviations

AIDS, acquired immuno - deficiency syndrome; AOR, adjusted odds ratio; COR, crude odds ratio; ETB, Ethiopian Birr; FGAE, Family Guidance Association of Ethiopia; FGD, focus group discussion; HIV, human immuno - deficiency virus; ICPD, International Conference on Population Development; IDI, in - depth interview; IHRERC, Institutional Health Research Ethics Review Committee; Km, kilometer; MPH, Masters of Public Health; NAYRHS, National Adolescent and Youth Reproductive Health Strategy; RH, reproductive health; RTIs, reproductive tract infections; SPSS, statistical software package for social sciences; SRH, sexual and reproductive health; STIs, sexually transmitted infections; VCT; voluntary counseling and testing; VIF, variance inflation factor; WHO, World Health Organization; YFS, youth friendly services

## Additional file

Additional file: Table S1. In-depth interview guide for service providers and youth in Harar town, Ethiopia, 2011. Table S2: Focus Group Discussion guide for youth in Harar town, Ethiopia, 2011. (DOCX 17 kb)
