# Supplementary material for: Utilization of youth friendly services and associated factors among youth in Harar town, east Ethiopia: a mixed method study
Source: BMC Health Serv Res. 2016 Jul 17;16:272. doi: 10.1186/s12913-016-1513-4 (PMC4947516; doi:10.1186/s12913-016-1513-4)
Supplement: Additional file: Table S1. — In-depth interview guide for service providers and youth in Harar town, Ethiopia, 2011. Table S2: Focus Group Discussion guide for youth in Harar town, Ethiopia, 2011. (DOCX 17 kb) [file 12913_2016_1513_MOESM1_ESM.docx]

**Table S1:** In-depth interview guide for service providers and youth in Harar town, Ethiopia, 2011.

| Experience And Training In Reproductive Health Services of staff provider | | | | | |
| --- | --- | --- | --- | --- | --- |
| Q1 | | I would like to ask you about the services you provide to youth. What services do you provide to client s of the age groups below at this health facility? (Check the appropriate boxes for each age group.) | | | |
|  | | | 10-14years | 15-19years | 20-24years |
| Contraceptive services | | |  |  |  |
| Antenatal care | | |  |  |  |
| Maternity care/delivery services | | |  |  |  |
| Postnatal care | | |  |  |  |
| HIV/AIDS counseling or IEC | | |  |  |  |
| HIV/AIDS testing (VCT) | | |  |  |  |
| Other STIs counseling or IEC | | |  |  |  |
| Other STI diagnosis | | |  |  |  |
| Treatment for incomplete abortions | | |  |  |  |
| Gynecological exams | | |  |  |  |
| Pregnancy testing | | |  |  |  |
| Reproductive health education | | |  |  |  |
| Others services | | |  |  |  |
| Q2. | How many years have you been  working in this health facility | | ---------------years  98 Don’t know | | |
| Q3 | How many years ago did you finish your basic training | | ----------------years  97No basic training  98 Don’t know | | |
| Q4 | Did your basic training cover these areas?(probe; do not read) | | 1.Contraceptive counseling  2.Antenatal Care  3.Maternity care/Delivery Services  4.Postnatal care  5.HIV/AIDS counseling or test  6.Treatment of incomplete abortion  7.Infertility counseling  8.Gynecological exams  9.Pregnancy testing  10.Reproductive health education | | |

| Q5 | Have you ever had refresher training in these areas?(Read and check if yes. For those areas checked yes, indicate the month and year of the last training. | Refresher Training? Check if yes | What was the month and year of your last refresher training? |
| --- | --- | --- | --- |
|  | 1. Contraceptive Counseling |  | ---------------/----------- |
|  | 1. Antenatal Care |  | ---------------/----------- |
|  | 1. Maternity care |  | ---------------/----------- |
|  | 1. Postnatal care |  | ---------------/----------- |
|  | 1. Treatment of incomplete abortions |  | ---------------/----------- |
|  | 1. Infertility consulting |  | ---------------/----------- |
|  | 1. Gynecological exam |  | ---------------/----------- |
|  | 1. Pregnancy testing |  | ---------------/----------- |
|  | 1. Reproductive health education |  | ---------------/----------- |
| Q6 | Have you attended any refresher or post basic training course especially on contraceptive clinical skills, or HIV/STI counseling, diagnosis and treatment? | 1.Yes  2.No  98 Don’t know skip Q28 |  |
| Q7 | How long was that training? | Months--------------years---------------- | |
|  | 1. HIV/AIDS training? | Month----------------years---------------  98Don’t know skip Q 28 | |
|  | 1. Learning about the special reproductive   needs of youth and special needs of youth | Month----------------years---------------  98Don’t know skip Q 28 | |
|  | C. Counseling youth | Month----------------years---------------  98Don’t know skip Q 28 | |
| Q8 | Are you friendly and respectful to youth clients If yes how? | 1 Yes  2 No |  |
| Q9 | Are you understanding and knowledgeable about youth concerns and needs? | 1 Yes  2 No |  |
| Q10 | Do you think ARH services are provided at convenient (and separate) hours for youth clients? | 1 Yes  2 No |  |
| **Thank You!** | | | |

**Table S2:** Focus Group Discussion guide for youth in Harar town, Ethiopia, 2011.

| Q1 | . What are your reasons for going to clinic? |
| --- | --- |
| Q2 | What is the reasons for not returning to the clinic? |
| Q3 | Are the services provided helpful to you? |
| Q4 | What do like and not like about service providers? |
| Q5 | We believe that youth have reproductive health rights. What does that mean to you? |
| Q6 | Do the service providers recognize that you have special needs as a young person? |
| Q7 | What additional services could they provide to meet your special needs as young person? |
| Q8 | What services are most helpful to you? |
| Q9 | In what ways could youth centers become a better place to visit for health and medical care for youth? |
| Q10 | What are the barriers you encounter to utilize youth-friendly services? |
| **Thank You** | |
